# Estern township Alzheimer Society, 40 years of development and collaboration for the patient and cargivers

**DOI:** 10.1002/alz70858_099511

**Published:** 2025-12-25

**Authors:** Guy Lacombe, Caroline Giguere, Julien Colin

**Affiliations:** ^1^ Sherbrooke University, Sherbbrooke, QC, Canada; ^2^ CIUSSS de l'Estrie‐CHUS, Sherbrooke, QC, Canada; ^3^ Societe Alzheimer de l'Estrie, Sherbrooke, QC, Canada; ^4^ Societe alzheimer de l''Estrie, Sherbrooke, QC, Canada

## Abstract

The Alzheimer Society of the Eastern Townships began 40 years ago with 6 volunteers bringing together professionals and caregivers to provide information to the families of people with Alzheimer's disease in the Sherbrooke region. As a non‐profit company, it was financed by donations from the public to pay for equipment and installation. It has gradually extended its services to the entire region covering more than 1100 square km and a population of 300,000 inhabitants. It now offers training to professionals and caregivers. Lectures by health professionals, lawyers and psychologists, monthly and open to the general public, cover the psychological, biological, social and legal components of Alzheimer's disease and related diseases. Day centres offer creative and enhancement activities in addition to music and pet therapy, no competent animators. In‐home respite services by trained staff help with home care. Supervised peer meetings reach both people with dementia and caregivers. A monthly newsletter is published and a dynamic but easy‐to‐access website is updated weekly. She collaborates with the Quebec Federation of Alzheimer and Alzheimer Societies Canada. The annual budget of a few thousand dollars now exceeds $1.5 million, partly supported by government subsidies for services not available in the public system. A house for temporary stays will soon be in operation

